# Silica nanoparticles promote wheat growth by mediating hormones and sugar metabolism

**DOI:** 10.1186/s12951-022-01753-7

**Published:** 2023-01-03

**Authors:** Yiting Li, Keyong Xi, Xi Liu, Shuo Han, Xiaowen Han, Gang Li, Lijun Yang, Dongfang Ma, Zhengwu Fang, Shuangjun Gong, Junliang Yin, Yongxing Zhu

**Affiliations:** 1 MARA Key Laboratory of Sustainable Crop Production in the Middle Reaches of the Yangtze River (Co-Construction By Ministry and Province), College of Agriculture, Yangtze University, Jingzhou, 434025 Hubei China; 2 Key Laboratory of Integrated Pest Management of Crops in Central China, Ministry of Agriculture/Hubei Key Laboratory of Crop Diseases, Institute of Plant Protection and Soil Science, Insect Pests and Weeds Control, Hubei Academy of Agricultural Sciences, Wuhan, 430064 Hubei China

**Keywords:** Photosynthesis, Chlorophyll, Plant hormone, Soluble sugar, Wheat, Growth

## Abstract

**Background:**

Silica nanoparticles (SiNPs) have been demonstrated to have beneficial effects on plant growth and development, especially under biotic and abiotic stresses. However, the mechanisms of SiNPs-mediated plant growth strengthening are still unclear, especially under field condition. In this study, we evaluated the effect of SiNPs on the growth and sugar and hormone metabolisms of wheat in the field.

**Results:**

SiNPs increased tillers and elongated internodes by 66.7% and 27.4%, respectively, resulting in a larger biomass. SiNPs can increase the net photosynthetic rate by increasing total chlorophyll contents. We speculated that SiNPs can regulate the growth of leaves and stems, partly by regulating the metabolisms of plant hormones and soluble sugar. Specifically, SiNPs can increase auxin (IAA) and fructose contents, which can promote wheat growth directly or indirectly. Furthermore, SiNPs increased the expression levels of key pathway genes related to soluble sugars (*SPS*, *SUS*, and α-*glucosidase*), chlorophyll (*CHLH*, *CAO*, and *POR*), IAA (*TIR1*), and abscisic acid (ABA) (*PYR*/*PYL*, *PP2C*, *SnRK2*, and *ABF*), whereas the expression levels of genes related to CTKs (*IPT*) was decreased after SiNPs treatment.

**Conclusions:**

This study shows that SiNPs can promote wheat growth and provides a theoretical foundation for the application of SiNPs in field conditions.

**Supplementary Information:**

The online version contains supplementary material available at 10.1186/s12951-022-01753-7.

## Background

Wheat (*Triticum aestivum* L.) is one of the most important cereal crops worldwide, ranking first terms of sowing area, production, and trade [1]. It is a staple food for approximately 40% of the world’s population [2]. Unfortunately, safe wheat production is threatened by diverse adverse stresses, including diseases, pests, salt, and drought [3]. Therefore, improving the resistance of wheat is crucial, and external application of substances such as nutrients and hormones is an economical and effective way to achieve this.

Silicon is the second most abundant element in the Earth’s crust and soil [4]. Although Si is not essential for the growth and development of most plants, it plays several positive roles in plant growth [5]. Currently, Si fertilizer is mainly applied to the soil by adding silicon materials such as blast furnace slag, so as to improve the soil and promote plant growth [6]. In recent years, nanomaterials have been widely used in agriculture as growth stimulators, plant protection products, and nano-fertilizers [7]. Among these, silica nanoparticles (SiNPs) have gained increasing attention because of their unique properties, such as high pore volume, large surface area, low toxicity, high stability, controllable morphology, and ease of surface functionalization [8].

The application of SiNPs to plants can effectively promote the photosynthesis and plant growth and improve their stress resistance [9]. Hussain et al. found that SiNPs application by the seed priming method can increase wheat biomass and yield through pot experiments [10]. However, the role of SiNPs in the growth and development of wheat has not been comprehensively studied in the field. In this study, we determined the effects of SiNPs on the growth of wheat, including growth parameters, chlorophyll content, net photosynthetic rate, endogenous hormones content, and soluble sugar content. These results suggest the beneficial effects of SiNPs on wheat grown in the field condition, which lays theoretical foundation for the application of SiNPs in crop field production.

## Results

### Silica nanoparticles deposition on wheat surface

**Fig. 1:**
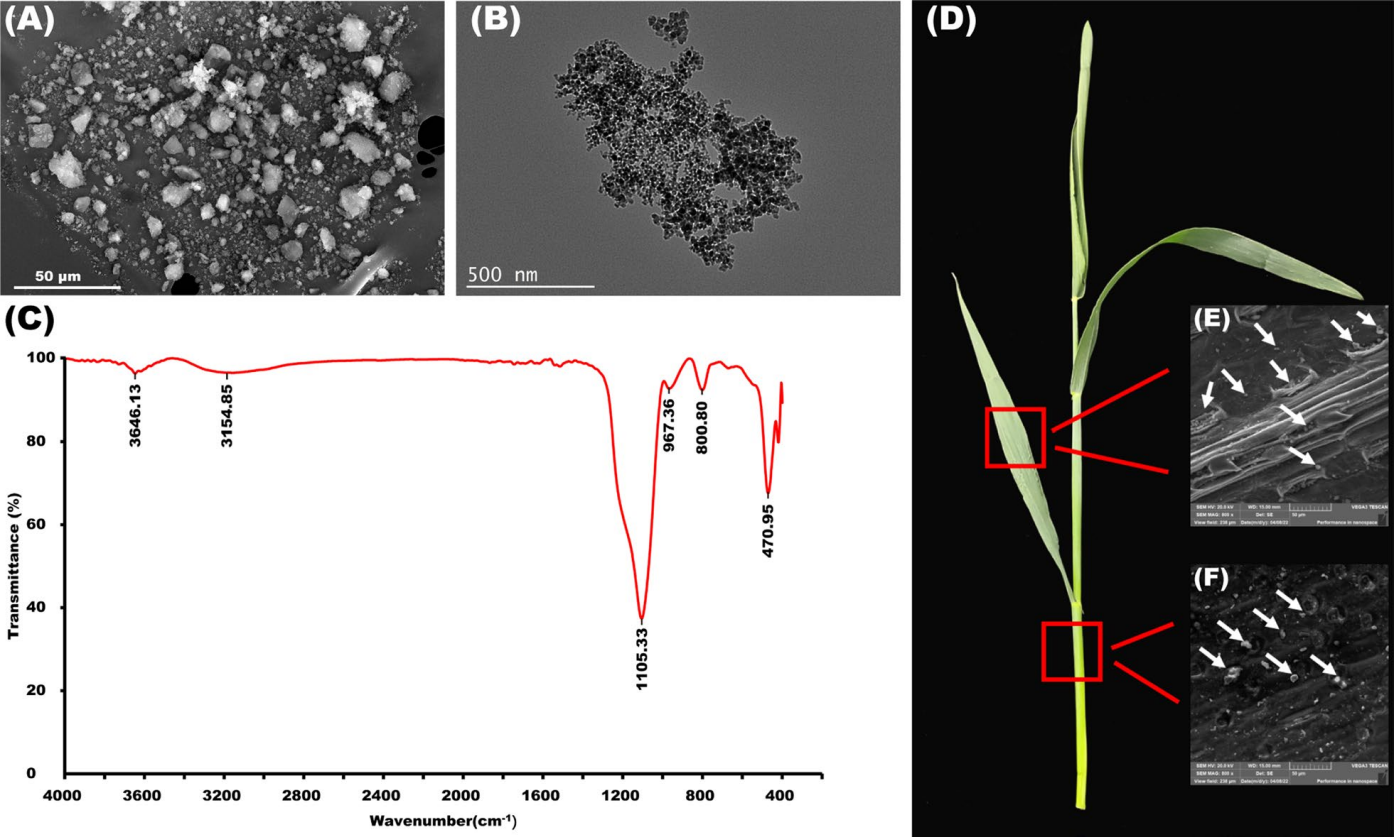
The SEM (**A**), TEM (**B**) micrographs and FTIR spectra (**C**) of SiNPs used in this study. (**D**) Deposition of silica nanoparticles on wheat leaves (**E**) and leaf sheaths (**F**) respectively. The white arrow represents the deposition position of SiNPs

To show the physical and chemical properties of the nanoparticles being used in this study, we performed SEM, TEM, and FTIR spectral analyses of silica nanoparticles (SiNPs) (Fig. 1). Their morphology characterized using SEM (Fig. 1A) and TEM (Fig. 1B) analyses revealed almost spherical nanoparticles. The FTIR spectra displayed broad peaks at 1105.33 (corresponding to the Si-O-Si) and 470.95 cm^−1^ (corresponding to the Si-O band) ranges (Fig. 1C). This indicates that the SiNPs used in this study were highly stable and pure. SiNPs were deposited on the surface of wheat leaves (Fig. 1E) and leaf sheaths (Fig. 1F), forming a mechanical barrier.

### SiNPs promoted wheat growth

**Fig. 2:**
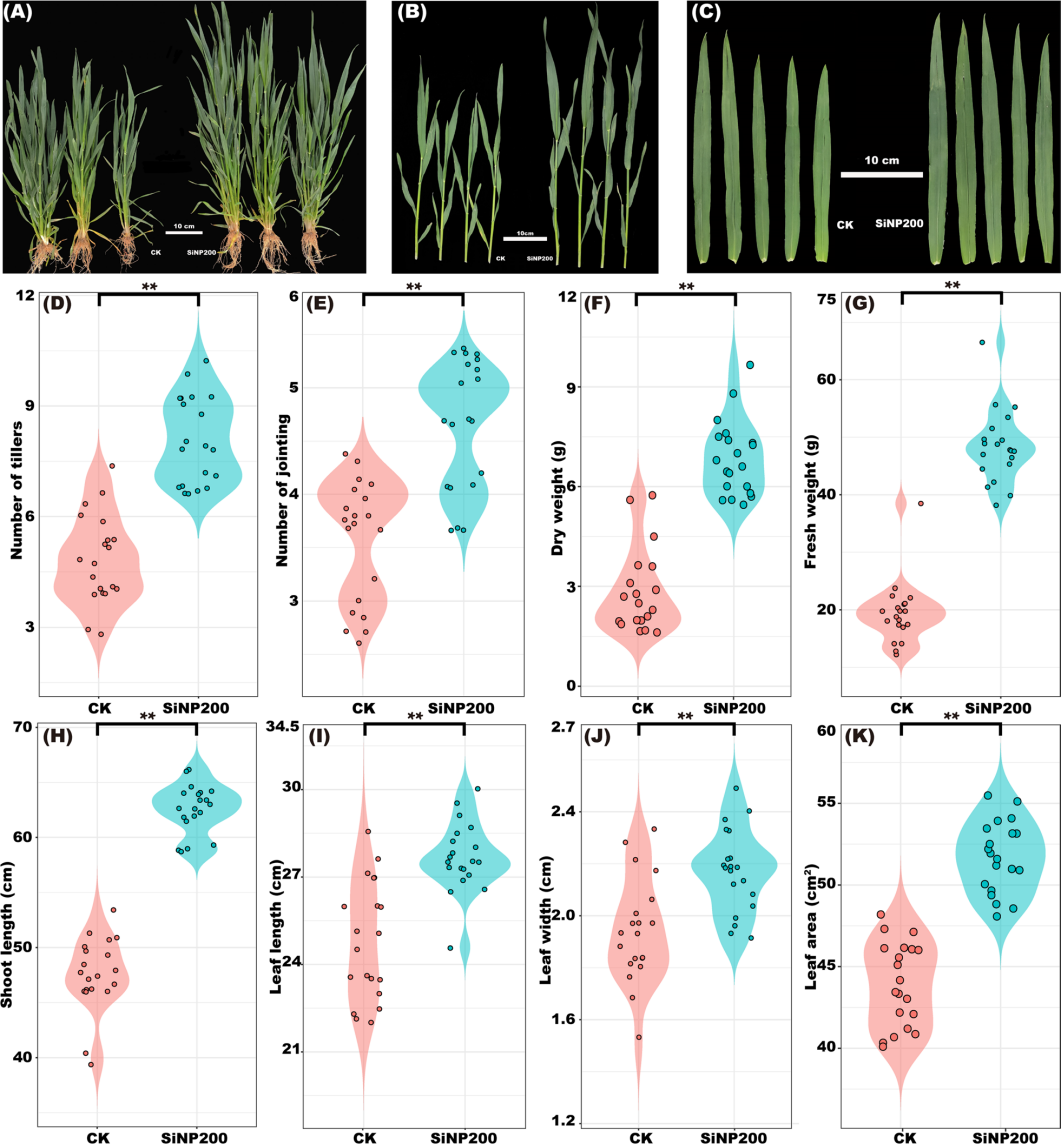
Effects of SiNP200 on wheat at jointing stage. Shoot length (**A**), stem length (**B**), and leaf length and width (**C**). Diagram of tillers (**D**), jointing (**E**), dry weight (**F**), fresh weight (**G**), shoot length (**H**), leaf length (**I**), leaf width (**J**), and leaf area (**K**). Values are means ± SE; n = 20 plants. **indicate the significant difference between the control and SiNP200 treatment at *p* < 0.01 (*t*-test)

The results showed that SiNP200 (200 mg·L^−1^ SiNPs) treatment improved the wheat growth and biomass in the field (Fig. 2A, B, C). As can been seen in Fig. 2D–K, compared with the control, the number of tillers, jointing, fresh weight, and dry weight of wheat increased by 66.7%, 27.4%, 142.7%, and 148.6%, respectively, and shoot length, leaf length, leaf width, and leaf area increased by 32.5%, 11.9%, 11.6%, and 17.8%, respectively.

### SiNPs promoted wheat growth by enhancing the photosynthesis

**Fig. 3:**
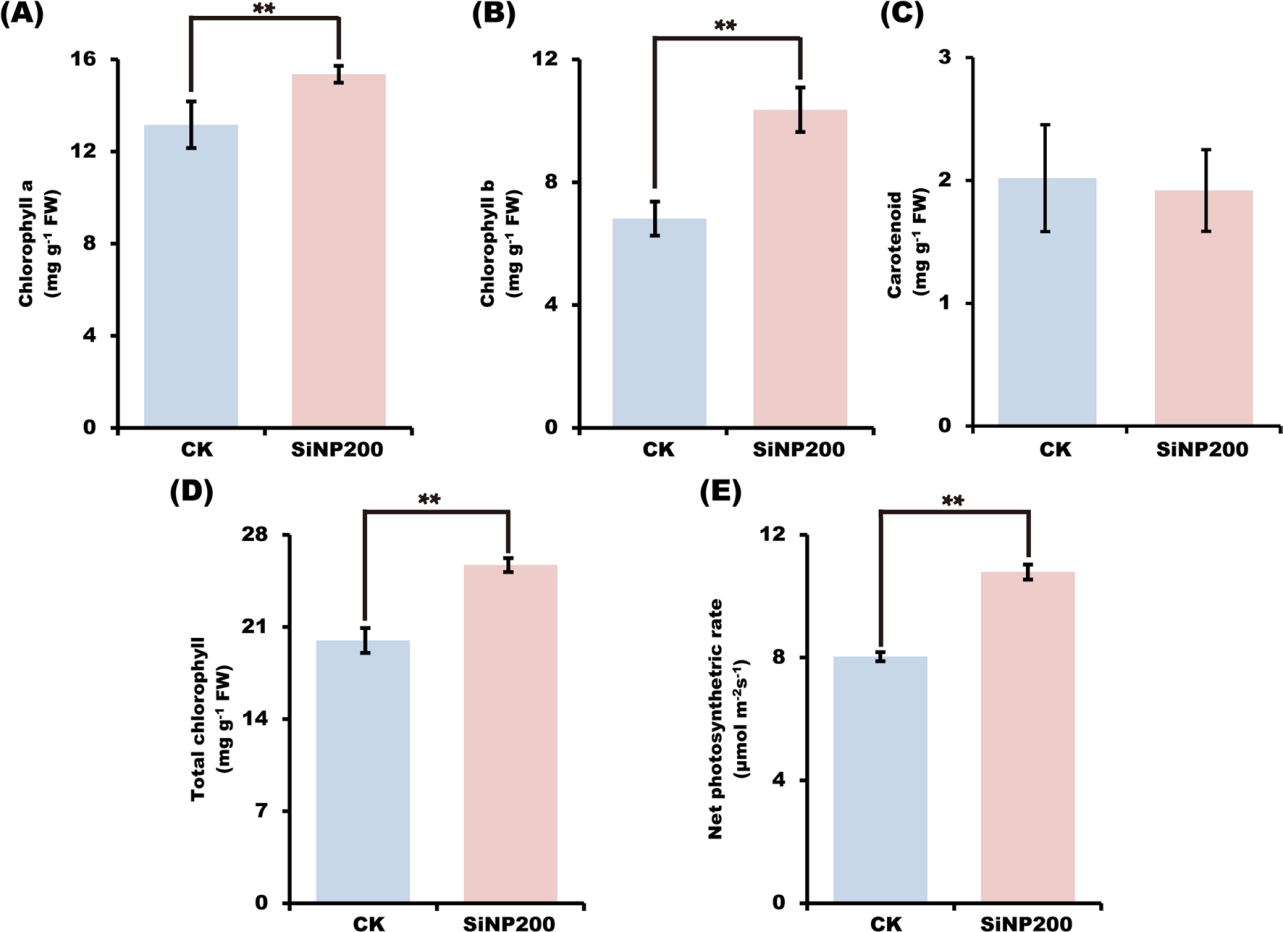
Effects of SiNP200 on the contents of chlorophyll a (**A**), chlorophyll b (**B**), carotenoid (**C**), total chlorophyll (**D**), and net photosynthetic rate (**E**) analysis. Values are means ± SE; n = 15. **indicate the significant difference between the control and SiNP200 treatment at *p* < 0.01 (*t*-test)

The results showed that SiNPs treatment increased the chlorophyll content in wheat leaves at the jointing stage (Fig. 3). Compared with the control, chlorophyll a and chlorophyll b contents increased by 1.17-fold and 1.52-fold, respectively. In contrast, the carotenoids content in the leaves decreased by 1.05-fold after the spraying with SiNPs. Furthermore, the net photosynthetic rate increased by 1.34-fold.

### SiNPs promoted wheat growth by regulating plant hormones

**Fig. 4:**
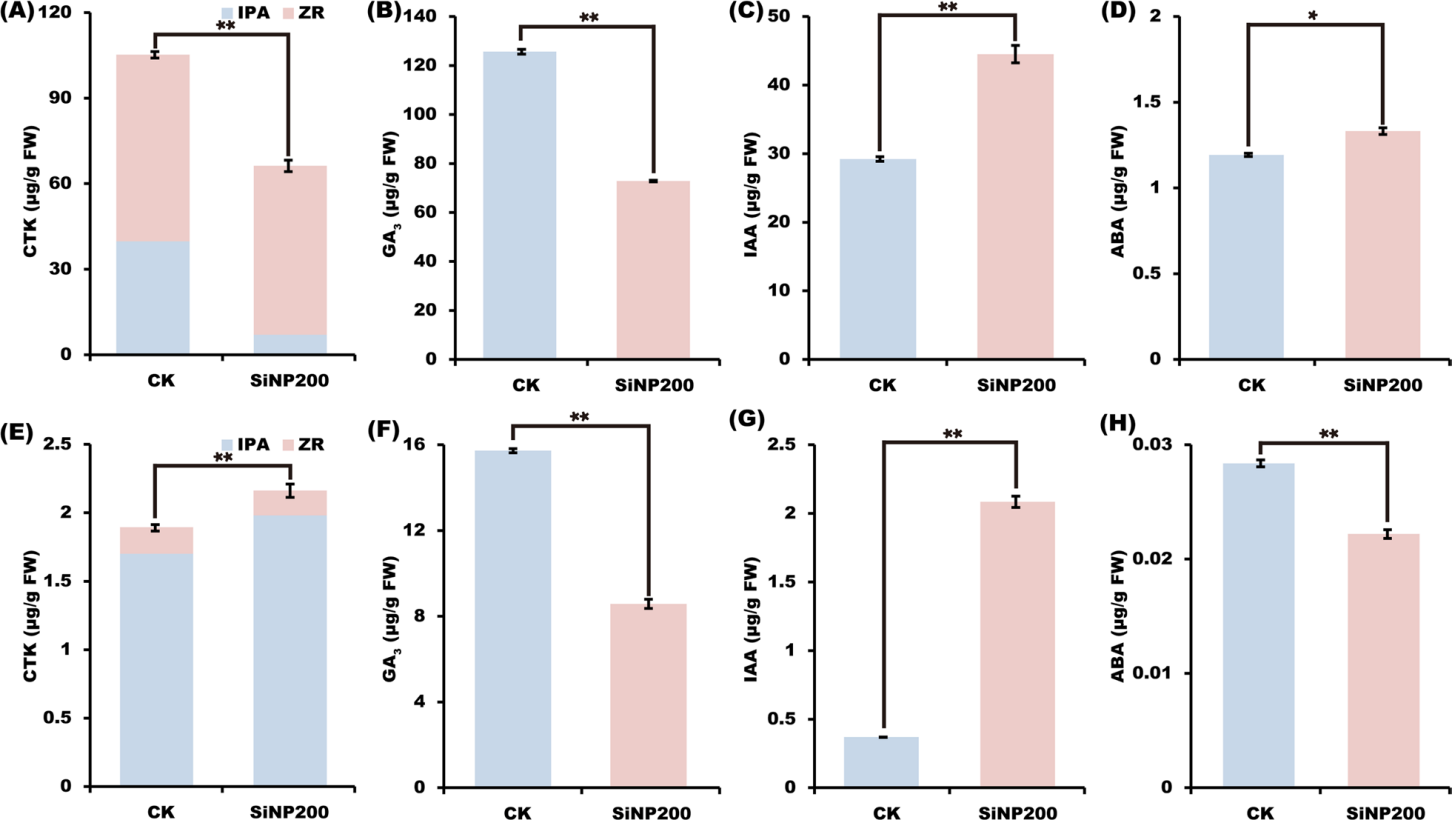
Effects of SiNP200 on the contents of CTK (**A**, **E**), GA_3_ (**B**, **F**), IAA (**C**, **G**) and ABA (**D**, **H**). (**A**–**D**) is wheat leaf sample; (**E**–**H**) is wheat stem sample. Values are means ± SE; n = 10. *, ** indicated that the significant difference between the control and SiNP200 treatment at *p* < 0.05 and *p* < 0.01, respectively (*t*-test)

As shown in Fig. 4, in the leaf, compared with the control, the contents of CTKs and GA_3_ were decreased by the SNP200 treatment, but the contents of auxin and abscisic acid were increased. In the stem, the contents of cytokinin and auxin were increased, but the contents of GA_3_ and ABA decreased after SNP200 treatment compared with the control.

### Soluble sugar content

**Fig. 5:**
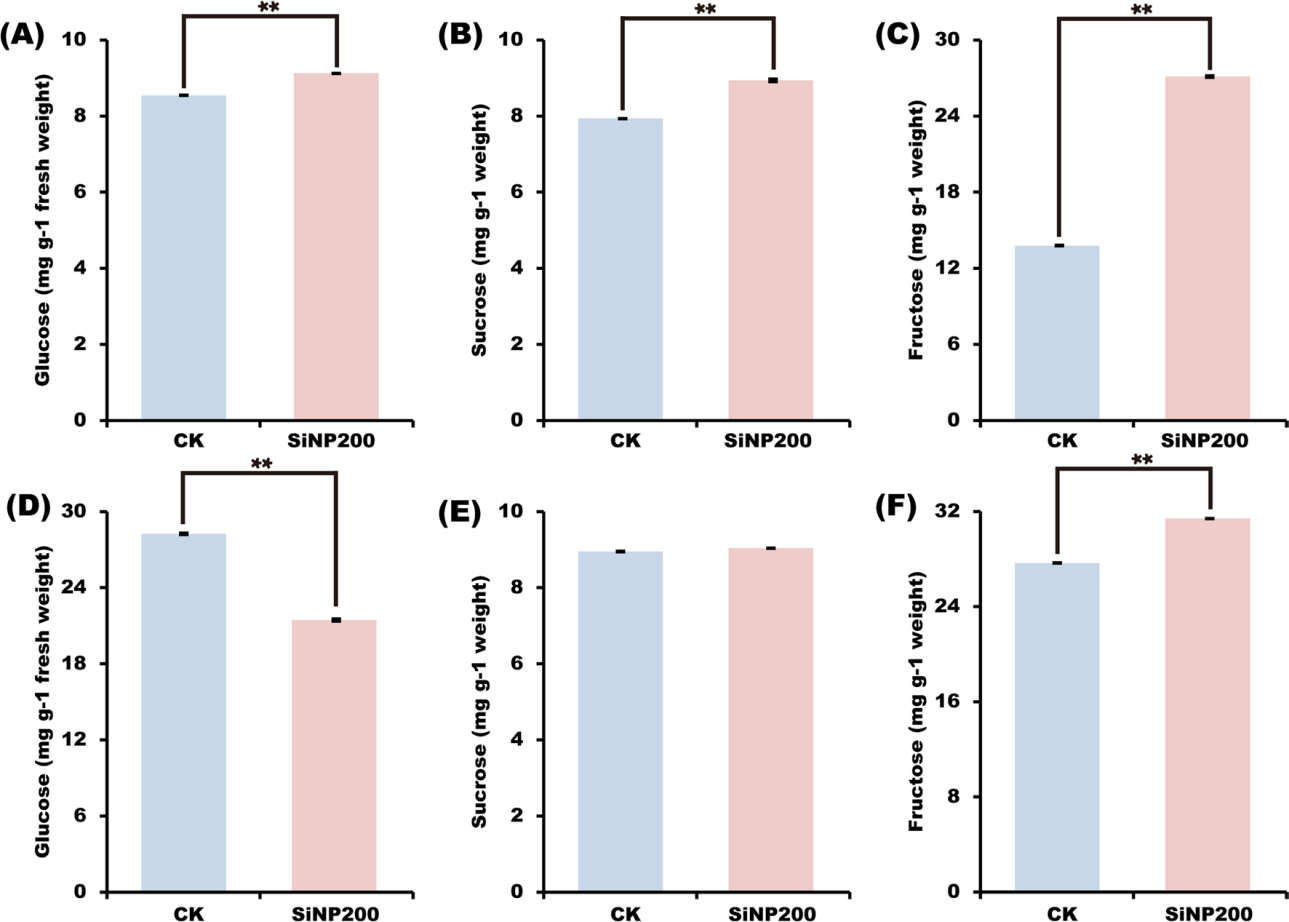
Effects of SiNP200 on the contents of glucose (**A**, **D**), sucrose (**B**, **E**), and fructose (**C**, **F**). (**A**–**C**) is wheat leaf sample; (**D**–**F**) is wheat stem sample. Values are means ± SE; n = 10. **indicate the significant difference between the control and SiNP200 treatment at *p* < 0.01 (*t*-test)

As shown in Fig. 5, in leaves, compared with the control, SiNPs increased glucose, sucrose, and fructose contents by 6%, 12.6%, and 96.4% at the jointing stage, respectively. In stems, compared with the control, the content of glucose was decreased by 26% after spraying with SiNPs, but sucrose and fructose contents were increased by 1% and 13.5%, respectively.

### RT-qPCR analysis of key pathway genes

**Fig. 6:**
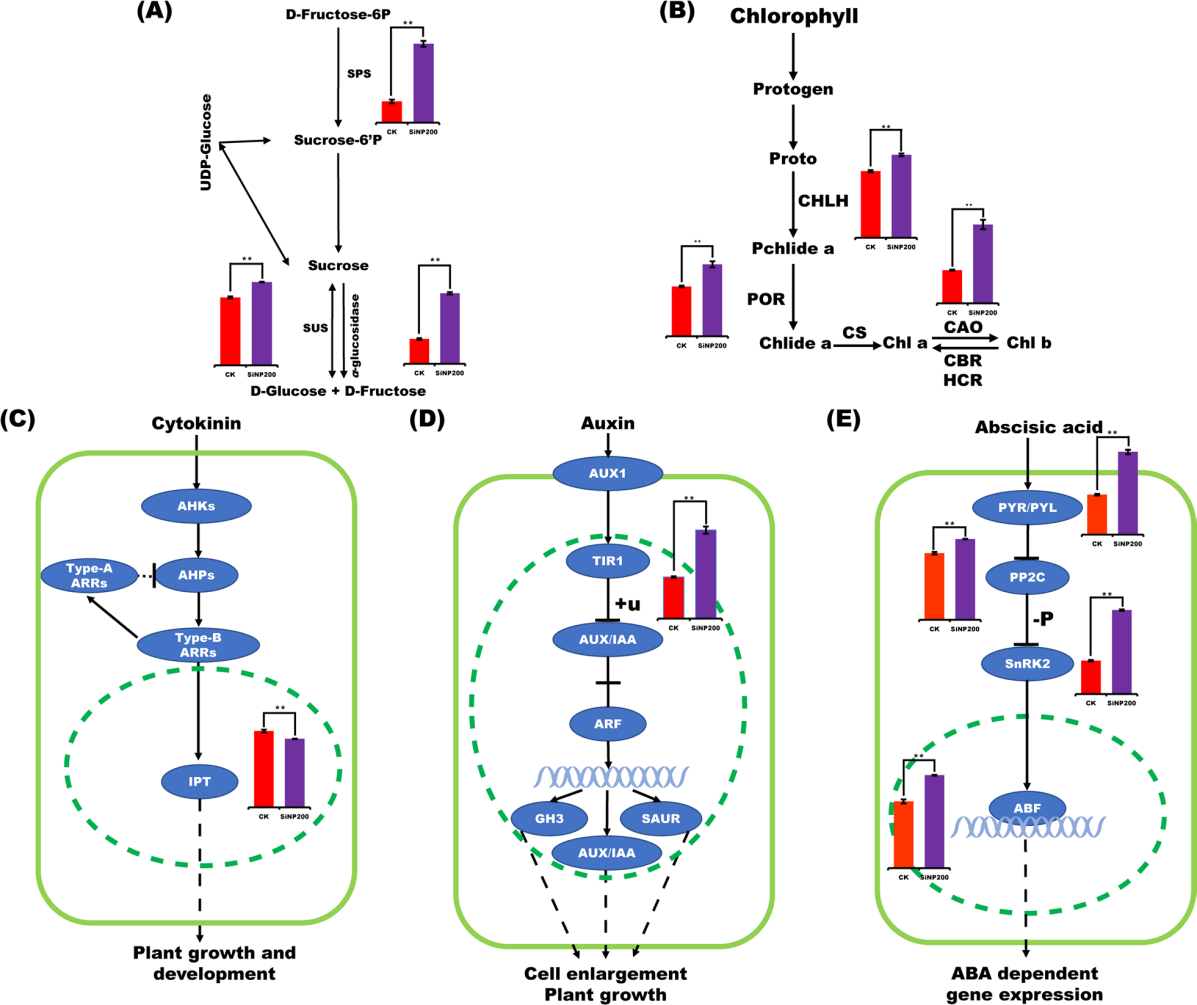
Soluble sugar (**A**), chlorophyll (**B**), CTKs (**C**), IAA (**D**), and ABA (**E**) synthesis and hydrolysis pathway and relative expression levels of related genes in wheat leaves. Values are mean ± SE of 3 replicates. **indicate the significant difference between the control and SiNP200 treatment at *p* < 0.01 (*t*-test)

*SPS* (sucrose-phosphate synthase) and *α*-*glucosidase* are the key enzymes for sucrose synthesis and decomposition, respectively, and *SUS* (sucrose synthase) is responsible for both [11]. In this study, the expression levels of *SPS*, *SUS*, and α-*glucosidase* genes in the leaves were up-regulated after SiNP200 treatment (Fig. 6A).

The expression levels of chlorophyll synthesis genes in the leaves, including *CHLH* (encoding Mg-chelatase), *POR* (encoding protochlorophyllide oxidoreductase), and *CAO* (encoding chlorophyllide an oxygenase), were all markedly up-regulated after SiNP200 treatment (Fig. 6B).

*IPT* (isopentenyl transferases) plays an important role in cytokinin biosynthesis [12]. In this study, compared with the control, the expression level of the *IPT* gene in leaves was down-regulated after SiNP200 treatment (Fig. 6C).

*TIR1* (TRANSPORT INHIBITOR RESPONSE), a receptor for auxin, is a member of an F-box protein group that contains five possible AFB proteins (AFB1–AFB5) that are crucial for substrate recognition in most phases of the auxin response [13]. Compared with the control, the expression level of the *TIR1* gene in leaves was up-regulated after SiNP200 treatment (Fig. 6D).

**Fig. 7:**
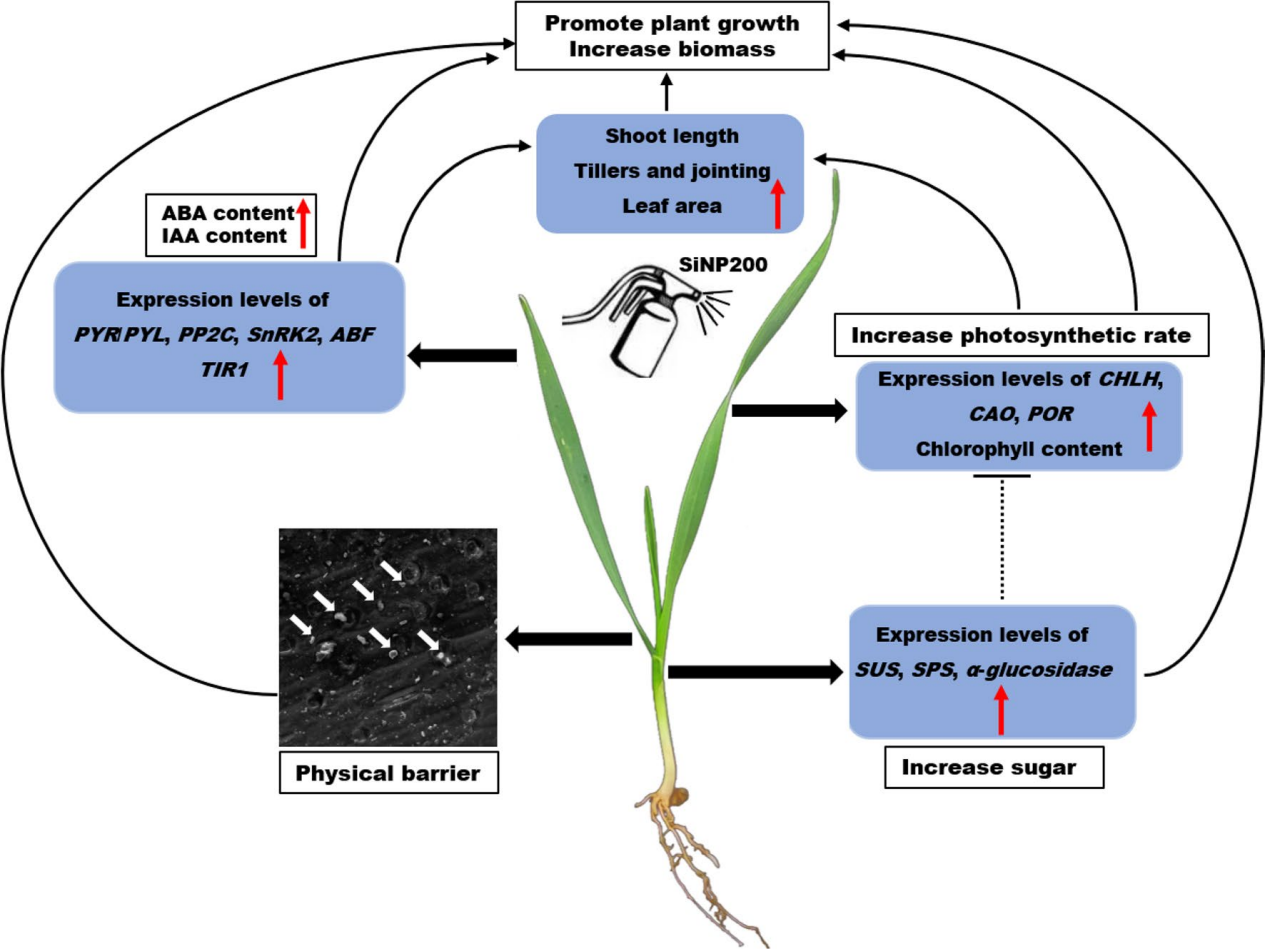
A possible model of the effect of SiNP200 treatment on wheat growth. Red arrows represent upregulation, and dashed lines represent possible but unconfirmed routes

The expression levels of genes involved in the ABA signal transduction pathway in leaves, including the PYR/PYL (PYRABACTIN RESISTANCE1/PYR1-LIKE), PP2Cs (type 2C protein phosphatases), SnRK2s (sucrose nonfermenting 1-related subfamily 2), and ABF, were all markedly up-regulated after SiNP200 treatment (Fig. 6E). Based on the results of RT-qPCR analysis and the change in the wheat growth index, we constructed a model of the effect of SiNP200 treatment on wheat (Fig. 7).

### Correlation and principal component analyses

**Fig. 8:**
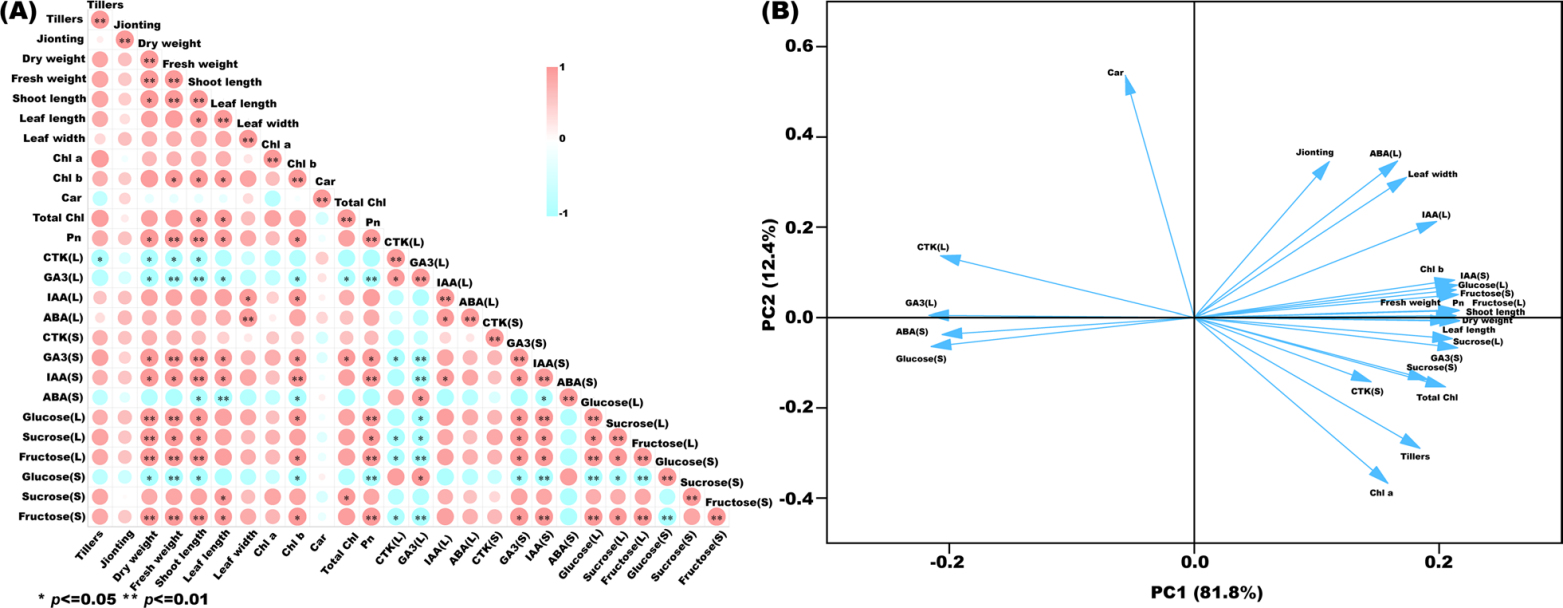
Correlation analysis and principal component analysis. **A** The heat map shows the correlation analysis between the observed parameters processed by SiNP200. **B** The score plot and loading plot shows the PCA analysis of the observation parameters processed by SiNP200. “L” represents leaf and “S” represents stem. * and ** denote correlation coefficients that are significant at *p* < 0.05 and 0.01 level, respectively (*t*-test)

The correlation of the parameters obtained in this study was analyzed, and the results are displayed as a correlation heat map (Fig. 8A). Dry weight has a significantly correlated with fresh weight, glucose (L), sucrose (L), fructose (L), and fructose (S) at *p* < 0.01 after SiNP200 treatment, and negatively correlated with CTK (L), GA_3_ (L), and glucose (S) at *p* < 0.05. Similarly, fresh weight has a significantly correlated with shoot length, Pn, GA_3_ (S), glucose (L), fructose (L), and fructose (S) at *p* < 0.01 after SiNP200 treatment, and correlated with Chl b, IAA (S), and sucrose (S) at *p* < 0.05, and has a negatively correlated with GA_3_ (L) and glucose (S) (*p* < 0.01). Shoot length has significantly correlated with Pn, GA3 (S), IAA (S), fructose (L), and fructose (S) (*p* < 0.01), and which has significantly negatively correlated with GA3 (L) (*p* < 0.01), CTK (L), ABA (S) and glucose (S) (*p* < 0.05) after SiNP200 treatment. Pn has a significantly correlated with IAA (S), glucose (L), fructose (L), and fructose (S) at *p* < 0.01 after SiNP200 treatment. The sum of the first two principal components treated with SiNP200 reached 94.2% (Fig. 8B). PC1 and PC2 accounted for 81.8% and 12.4% of the total variance, respectively.

## Discussion

Si has been proven to play a favorable role in plant growth, mineral nutrition, mechanical strength, and resistance to fungal diseases [14, 15]. To investigate the effect of nanoparticles on plants, the nanoparticles are usually prepared as suspensions and sprayed onto plants or by soaking the plants into suspension [7, 16, 17]. In this study, a SiNP200 suspension was prepared and sprayed onto the leaves. As reported by Merwad et al. [18] and Fawe et al. [19] reported that Si is deposited on the surface of gramineous plants and induces a mechanical physical barrier. Similarly, in this study, we observed the deposition of SiNPs on the surface of wheat leaves. Furthermore, SiNP200 application significantly enhanced the wheat biomass and growth, which may be due to SiNPs forming a physical barrier in wheat leaf tissue to protect wheat from natural environmental stresses such as drought, and heat during growth and development, thus promoting wheat growth [20]. However, we did not detect SiNPs in the tissues of the studied plants. As reported by El-Shetehy et al. [21], direct TEM imaging of nanoparticles in Arabidopsis leaves showed that numerous nanoparticles accumulated around the stomata and entered the plant not only through the stomata but also through the nearby epidermal cells. However, the manner in which nanoparticles enter wheat leaves should be further studied, and their amount and accumulation status in tissues should also be determined. Another reason may be that the enhanced nutrient supply to plants by SiNPs application, as suggested by Fraceto et al. [22].

Chlorophyll content is a key factors in determining the intensity of photosynthesis and biomass accumulation [23]. A previous study showed the positive effect of silica nanoparticles in salt-stressed tomato by improving the photosynthesis rate, mesophyll conductance, and water use efficiency [24]. Additionally, SiNPs can reportedly induce synthesis of chlorophyll in *Larix olgensis* [25]. In the present study, SiNPs increased the expression levels of chlorophyll synthesis genes, which may contribute to the increased chlorophyll a and b contents in wheat leaves. In rice, the accumulation of SiNPs in leaves is beneficial for maintaining leaves upright and stretching leaf surfaces to capture maximum sunlight, thus promoting photosynthesis [26]. In this study, we found that SiNPs could increase leaf area by 17.8% compared with the control, which may also contribute to enhancing the photosynthesis and therefore, biomass.

Nanoparticles can widely affect the photosynthesis. For example, in wheat, Dias et al. [27] found that titanium dioxide nanoparticles (TiO_2_-NPs) decreased the chlorophyll a contents and disturbed stomatal conductance, thus compromising the electron transport through PSII and impairing CO_2_ assimilation. Interestingly, in spinach, Gao et al. [28] and Zheng et al. [29] found that TiO_2_-NPs improved the activity of the Rubisco enzyme, thereby strengthening Rubisco carboxylation and increasing the rate of photosynthesis, thus promoting the growth. Li et al. [30] revealed that chromic oxide nanoparticles (Cr_2_O_3_NPs) could damage the chloroplast ultrastructure of soybean, causing irreversible damage and reducing photosynthesis. Abdel-Aziz and Rizwan [31] suggested that in bean seedlings, silver nanoparticles (AgNPs) resulted in irregular shape of chloroplasts, formation of protrusions and extensions, and less stacking of Greene. In contrast, in this study, we found that SiNP200 increased chlorophyll contents, which may contribute to the increased net photosynthetic rate (Fig. 3E). The underlying reasons could be altered chloroplast ultrastructure, stomatal conductance, Rubisco enzyme activity, and so on, which need to be further investigated.

Endogenous hormones are signals that play important roles in seeds germination and tiller bud growth and plant growth [32, 33]. Cytokinins (CTKs) regulate plant growth and developmental processes by regulating cell proliferation and differentiation [34]. In salt-stressed tomato, Si (Na_2_SiO_3_•9H_2_O) addition increased the CTKs levels in the leaves [35]. In wheat, CTKs play an important role in the regulation of tiller bud growth [36]. The external application of CTKs has been reported to stimulates tiller bud growth in wheat (*Triticum aestivum* L.). During the jointing stage, the main function of CTKs is to promote internode elongation [32]. Therefore, in this study, SiNPs decreased the expression level of the *IPT* gene and content of CTKs in wheat leaves, but increased their content in the stems at the jointing stage (Fig. 5A, E), which helped to increase the stem length of wheat after SiNPs treatment (Fig. 2B).

As an important hormone in plants, gibberellin participates in the regulation of many developmental processes, such as seed development, stem elongation, leaf stretching, and flower induction. In wheat, both paclobutrazol and GA_3_ application increased lodging resistance of winter wheat. GA_3_ application significantly increased the plant height and length of the second basal internode, and altered lignin accumulation and its related enzymes activity in the basal internode, whereas paclobutrazol had the opposite effect [37]. In the present study, SiNPs increased the height but decreased GA_3_ in wheat leaves. The GA_3_ content needs to be detected in more tissues—for example, the stem, internode, and root, and more growth period (stem elongation stage, milk-filling stage) to confirm the changes in GA_3_ in response to SiNPs treatment. In addition, because this experiment was conducted under field conditions, plant hormones were easily affected by environmental factors such as temperature. SiNPs-induced changes in hormone levels need to be confirmed in the laboratory under stable environmental conditions.

Auxin (IAA) is mainly produced in vigorous growth parts and has a positive effect on promoting cell elongation and organ differentiation [13]. After spraying SiNPs, the expression level of the *TIR1* gene and IAA content in wheat leaves and stems increased (Fig. 5C, G), which potentially contributed to the promoting wheat growth with SiNPs addition (Fig. 2).

ABA plays an important role in growth and development under non-stressed conditions, and regulates various aspects of cellular growth, including cell division, enlargement, differentiation, and central metabolism [38]. In this study, SiNPs increased the expression levels of genes related to ABA signal transduction and content, which could contribute to promoting wheat growth. These results imply that SiNPs may promote wheat growth by regulating the metabolism of endogenous hormones.

Sugars are the main products of photosynthesis and are well known as energy providers and building blocks of various structural components of plant cells, tissues, and organs [39]. Additionally, as part of various sugar signaling pathways, they interact with other cellular machinery and influence many important cellular processes in plants [40]. Mahakham et al. found that priming the rice seeds with AgNPs increased the biosynthesis of total soluble sugars [41]. Here,the SiNPs treatment increased the content of soluble sugars in the leaves (Fig. 6A–C).

Based on the results of previous studies, the reason for soluble sugars content being increased by SiNPs may be partly due to enhanced photosynthesis promoting the synthesis of soluble sugars [42]. Soluble sugar is mainly synthesized in leaves and transported to roots and panicles through the stem, where it can store a certain amount of soluble sugar for later grain filling [43], which may be the reason for the increase in sucrose and fructose content in the stem. SPS, *α*-glucosidase, and SUS are key enzymes involved sucrose synthesis and decomposition [11]. The expression levels of *SPS*, *SUS*, and *α*-*glucosidase* increased after SiNPs treatment, which is of substantial significance for the enhanced sugars content.

We used cv. Changnuomai 1 to determine the infield effects of SiNPs on wheat plants. Notably, only a single wheat genotype was identified in a single environment. Some genotypes that carry alleles, such as *Rht-B1b*, may have impacted these results. We then analyzed the genotypes and found that Changnuomai 1 contained both *Rht-B1b* and *Rht-D1b* (Additional file 2: Fig. S1). According to the results of Liu et al. [44] 60.1% and 42.1% of Chinese wheat cultivars contain *Rht-B1b* and *Rht-D1b*, respectively, and 24.3% of cultivars contain both *Rht-B1b* and *Rht-D1b*. Therefore, it seems that cv. Changnuomai 1 is a representative genotype and in the future, more genotypes should be tested in multiple environments to confirm the effects of SiNPs on wheat growth. Meanwhile, for practical field production, growers are fairly concerned about the harvest index, lodging, and phenology. Although we observed that the ripening stage was approximately one week earlier than the control and the height of plants was not significantly different at the ripening stage, reliable data are still lacking to draw conclusions about harvest and quality traits. In the future, we will systematically determine the harvest and seed quality traits, including the content of wet gluten, stable time of dough, sedimentation value, and starch and protein contents.

## Conclusion

Our results showed that spraying SiNPs on wheat leaves promoted the growth and improved the physiological indices of wheat. SiNPs treatment also improved wheat photosynthesis, promoted sugar synthesis and transport, and increased the endogenous hormone level in wheat. However, it should also be noticed that transcript or hormone and sugar levels may not be representative of the overall effects of the SiNPs under in this study. More evidence is needed to confirm these results in the future studies. Nevertheless, these results indicate that silica nanoparticles can be used as a supplements for chemical fertilizers to improve wheat performance in the field.

## Methods

### Plant materials and treatment

Wheat seeds (*Triticum aestivum* L. cv. Changnuomai 1) were provided by the Laboratory of Crop Genetics and Breeding, College of Agriculture, Yangtze University, China. The *Rht* genotype of this cultivar was analyzed according to the method reported by Ellis et al. [45], where wheat seeds of the same size were first disinfected with 5% (v:v) sodium hypochlorite solution for 10 min and then rinsed with sterilized distilled water for five times [46]. Silica nanoparticles (SiNPs, E551 food additive) were purchased from Sigma-Aldrich (Lot 637238, the purity is 99.5%, and particle size is 10–20 nm). The concentration of the SiNPs solution was selected based on the study by Alsaeedi et al. [47] and our preliminary experiments. The SiNPs (200 mg L^−1^) were suspended in deionized water by sonicating the silica bundles using an ultrasonicator at 10 MHz for ∼40 min, resulting in a partially homogeneous solution (SiNP200) [17]. SiNPs characterized by scanning electron microscopy (SEM, Hitachi S4160, Japan), transmission electron microscopy (TEM, LEO 906E, Zeiss, Germany), and Fourier Transform infrared spectroscopy (Spectrum 400, Perkin Elmer, USA). The above sterilized wheat seeds were evenly divided into two groups.

The seeds were treated as follows: (1) control (CK), seeds were dipped in distilled water for 1 h at room temperature; (2) SiNP200, seeds were immersed into 200 mg L^−1^ SiNPs for 1 h at room temperature. After air-drying, the two groups of wheat seeds were sown separately on November 12, 2021; the number of grains in each row was fixed at 100, and the spacing was as consistent as possible. Starting on December 12, 2021 (the beginning of the wintering period), the wheat leaves were sprayed with SiNP200 for three times, once a day; and from February 19, 2022 (the beginning of the regreening stage), the SiNP200 suspension was sprayed on the leaves three times, once each day. The experiment was performed using a completely randomized design with three replicates. Leaf and stem samples were collected for physiological and biochemical studies on March 14, 2022 (jointing stage). Wheat growth stages were divided according to the research of Zadoks et al. [48].

### Scanning Electron Microscope (SEM) analysis

Leaves and leaf sheaths samples were prepared for SEM analysis as follows, first, the samples were dried for 30 min using a freeze-dryer (BoYiKang FD-1A-50, Beijing, China), sputter-coated with gold at 12 Ma and 1.5 kV using a Coater (Ion Sputter JFC-1100, Tokyo, Japan), and then Scanning Electron Microscopy (SEM, VEGA3.TESCAN) was used to observe the SiNPs deposition on the surface of the sample.

### Measurement of growth parameters

The plant height, leaf length, and leaf width were measured during the jointing stage. The tiller and jointing numbers were then counted. The plants were washed with deionized water, gently dried on blotting paper, and weighed (fresh weight). The samples were then dried in an oven at 70 °C for 48 h, and the dry weight was measured. Each treatment group consisted of 20 seedlings.

### Determination of net photosynthetic rate and chlorophyll content

The second leaf from the top was used to measure chlorophyll content and determine the net photosynthetic rate. The net photosynthetic rate was measured using portable photosynthetic fluorimeter (Yaxin-1102, Beijing, China) on a sunny day (between 10 and 11 am). For chlorophyll determination, 0.1 g fresh tissue from the second leaf was soaked in 10 mL anhydrous ethanol and incubated at 25 ℃ for 24 h in the dark. After the green color of the leaves faded, the chlorophyll content was determined using ethanol colorimetry. The absorbance was measured at 665, 649, and 470 nm, respectively. Chlorophyll concentration was calculated according to the following formula (mg g^−1^): chlorophyll a = 13.95 OD_665_-6.88 OD_649_, chlorophyll b = 24.96 OD_649_-7.32 OD_665_, carotenoid = (1000 OD_470_-2.05 Chl a-111.48 Chl b)/245 [49, 50].

### Determination of endogenous hormones

Fresh leaves and stems were taken for endogenous hormones determination, ground with liquid nitrogen, weighed to 0.2 g, placed them into a 1.5 mL centrifuge tube, and stored them in a refrigerator at – 80 ℃ until use. The hormone content, including abscisic acid (ABA), gibberellin (GA_3_), auxin (IAA) and cytokinin (zeatin nucleoside ZR, isopentenyl adenine nucleoside IPA), was determined using the HPLC-UV external standard method, which was completed by Suzhou Gris Biotechnology Co., Ltd (Jiangsu, China).

### Determination of soluble sugar

Soluble sugars, including glucose, sucrose, and fructose, were determined using the kits purchased from Suzhou Gris Biotechnology Co., Ltd (Jiangsu, China). The kits were used in accordance with the manufacturer’s instructions.

### Gene expression analysis

Leaf samples were collected at the jointing stage and used for quantitative real-time polymerase chain reaction (qRT-PCR) analysis. Total RNA in the leaves was extracted using the Trizol reagent (GenStar, Beijing, China) and digested with DNaseI (TaKaRa, Dalian, China) to remove DNA. The RNA was reverse transcribed to complementary DNA (cDNA) using RevertAid Reverse Transcriptase (Vazyme, Nanjing, China). The quantitative real-time polymerase chain reaction (qRT-PCR) analysis was performed on a CFX 96 Real-time PCR system (Bio Rad, Hercules, CA, USA) using SYBR Green Master Mix (Vazyme, Nanjing, China) according to the manufacturer’s instructions. Gene-specific primers were designed using Primer Premier 5 software and are listed in Additional file 1: Table S1. *TaActin* (Forward: ATGGAAGCTGCTGGAATCCAT, Reverse: CCTTGCTCATACGGTCAGCAATAC) was used as an internal reference gene for qRT-PCR analysis [51].

### Statistical analysis

All experiments and determinations were conducted at least in triplicate. The data from all experiments are expressed as the means ± SE. Student’s *t*-test was used to determine significant differences between the means using SPSS 20.0 (IBM Co., Armonk, NY, USA). Principal component analysis (PCA), correlation analysis, and figures were prepared using Origin 2021 (OriginLab Co., Northampton, MA, USA).

## Supplementary Information

**Additional file 1: Table S1.** The primer sequences for qRT-PCR.

**Additional file 2: Figure S1.** *Rht* genotype analysis of wheat cv. Changnuomai 1.

## Data Availability

All data generated or analyzed during this study are included in this manuscript and its Additional files.
