# Supplementary material for: Silica nanoparticles promote wheat growth by mediating hormones and sugar metabolism
Source: J Nanobiotechnology. 2023 Jan 3;21:2. doi: 10.1186/s12951-022-01753-7 (PMC9808955; doi:10.1186/s12951-022-01753-7)
Supplement: Supplementary file 2 — Additional file 2: Figure S1. Rht genotype analysis of wheat cv. Changnuomai 1. [file 12951_2022_1753_MOESM2_ESM.docx]

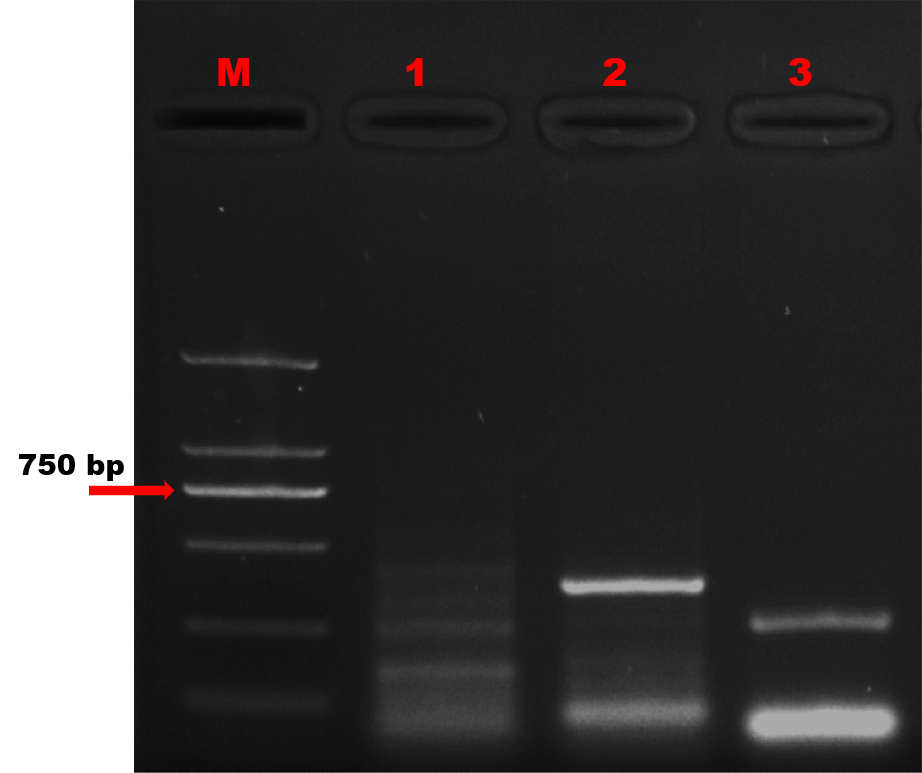


**Supplementary Fig. 1.** *Rht* genotype analysis of wheat cv. Changnuomai 1. PCR products were separated on 1.5% agarose gels. M. 2000 bp DNA ladder, 1. PCR product amplified with the primer set of NH-BF.2 and WR1.2 for *Rht-B1a* gene, 2. PCR product amplified with the primer set of NH-BF.2 and MR1 for *Rht-B1b* gene, 3. PCR product amplified with the primer set of DF and MR2 for *Rht-D1b* gene.
